# *AtCesA8*-driven *OsSUS3* expression leads to largely enhanced biomass saccharification and lodging resistance by distinctively altering lignocellulose features in rice

**DOI:** 10.1186/s13068-017-0911-0

**Published:** 2017-09-16

**Authors:** Chunfen Fan, Shengqiu Feng, Jiangfeng Huang, Yanting Wang, Leiming Wu, Xukai Li, Lingqiang Wang, Yuanyuan Tu, Tao Xia, Jingyang Li, Xiwen Cai, Liangcai Peng

**Affiliations:** 1 Biomass and Bioenergy Research Centre, Huazhong Agricultural University, Wuhan, China; 2 National Key Laboratory of Crop Genetic Improvement, Huazhong Agricultural University, Wuhan, China; 3 College of Plant Science and Technology, Huazhong Agricultural University, Wuhan, China; 4 College of Life Science and Technology, Huazhong Agricultural University, Wuhan, China; 5 HaiKou Experimental Station, Chinese Academy of Tropical Agricultural Sciences, Haikou, 570102 China; 6 Department of Plant Science, North Dakota State University, Fargo, ND USA

**Keywords:** Sucrose synthase, Transgenic rice, Biomass saccharification, Yeast fermentation, Lodging resistance, Cell wall, Cellulose crystallinity

## Abstract

**Background:**

Biomass recalcitrance and plant lodging are two complex traits that tightly associate with plant cell wall structure and features. Although genetic modification of plant cell walls can potentially reduce recalcitrance for enhancing biomass saccharification, it remains a challenge to maintain a normal growth with enhanced biomass yield and lodging resistance in transgenic plants. Sucrose synthase (SUS) is a key enzyme to regulate carbon partitioning by providing UDP-glucose as substrate for cellulose and other polysaccharide biosynthesis. Although *SUS* transgenic plants have reportedly exhibited improvement on the cellulose and starch based traits, little is yet reported about *SUS* impacts on both biomass saccharification and lodging resistance. In this study, we selected the transgenic rice plants that expressed *OsSUS3* genes when driven by the AtCesA8 promoter specific for promoting secondary cell wall cellulose synthesis in *Arabidopsis*. We examined biomass saccharification and lodging resistance in the transgenic plants and detected their cell wall structures and wall polymer features.

**Results:**

During two-year field experiments, the selected AtCesA8::*SUS3* transgenic plants maintained a normal growth with slightly increased biomass yields. The four independent transgenic lines exhibited much higher biomass enzymatic saccharification and bioethanol production under chemical pretreatments at *P* < 0.01 levels, compared with the controls of rice cultivar and empty vector transgenic line. Notably, all transgenic lines showed a consistently enhanced lodging resistance with the increasing extension and pushing forces. Correlation analysis suggested that the reduced cellulose crystallinity was a major factor for largely enhanced biomass saccharification and lodging resistance in transgenic rice plants. In addition, the cell wall thickenings with the increased cellulose and hemicelluloses levels should also contribute to plant lodging resistance. Hence, this study has proposed a mechanistic model that shows how *OsSUS3* regulates cellulose and hemicelluloses biosyntheses resulting in reduced cellulose crystallinity and increased wall thickness, thereby leading to large improvements of both biomass saccharification and lodging resistance in transgenic rice plants.

**Conclusions:**

This study has demonstrated that the AtCesA8::*SUS3* transgenic rice plants exhibited largely improved biomass saccharification and lodging resistance by reducing cellulose crystallinity and increasing cell wall thickness. It also suggests a powerful genetic approach for cell wall modification in bioenergy crops.

**Electronic supplementary material:**

The online version of this article (doi:10.1186/s13068-017-0911-0) contains supplementary material, which is available to authorized users.

## Background

Crop biomass residues represent enormous lignocellulose resource for the production of biofuels and chemicals [1]. Currently, lignocellulosic ethanol is considered as a promising short-term alternative to fossil fuels, due to its abundance in the cropping systems and having no conflict between energy demand and food supply [2, 3]. The production of lignocellulosic ethanol involves three major steps, including pretreatments for cell wall destruction, enzymatic hydrolysis for sugar release, and yeast fermentation for ethanol production [4, 5]. Because lignocellulose recalcitrance is a major hindrance for biomass conversion, genetic modification of plant cell walls has been proposed for enhancing biomass saccharification. However, plant cell walls have complicated structures and diverse functions, and their modification may thus affect plant growth and mechanical strength, in particular on plant lodging resistance. Since lodging is a major integrated trait tightly associated with grain and biomass yields in cereal crops [6–8], it becomes important to enhance both biomass saccharification and lodging resistance in transgenic plants.

Plant cell walls are mainly composed of cellulose, hemicelluloses, and lignin with small amounts of pectin and wall proteins. In principle, the wall polymers form a complex crosslink network with recalcitrant property against biomass saccharification [9–11]. In addition, the quantity and features of wall polymers are closely associated with plant lodging resistance [12–18]. For instance, cellulose crystallinity has been reported as the key factor that negatively affects either the biomass enzymatic digestibility or the plant lodging resistance [19]. In addition, the hemicelluloses’ contents have been shown to negatively affect cellulose crystallinity, probably due to their association with cellulose microfibrils via hydrogen bonds [5, 19–21]. Hence, genetic modification of plant cell walls may be a promising solution to both biomass recalcitrance and plant lodging [9, 22–25].

Cellulose is the major component of plant cell walls and provides fermentable glucose for bioethanol production. In plants, cellulose synthase (CESA) catalyzes cellulose biosynthesis using UDP-glucose (UDPG) as substrate [26]. Sucrose synthase (SUS) catalyzes reversible conversion of sucrose and UDP into UDPG and fructose [27–29]. Importantly, SUS has been characterized to regulate cellulose biosynthesis by providing UDPG substrates in cotton fibers [30]. Although *SUS* transgenic plants have reportedly exhibited improvement in the cellulose-based traits [30–35], little is known about SUS impact on plant lodging resistance.

Rice is a major food crop around world with large amounts of biomass residues for biofuel production. In this study, we selected transgenic rice plants that overexpressed *OsSUS3* gene using *Arabidopsis* cellulose synthase (AtCesA8) gene’s promoter, specific for secondary cell wall synthesis. We detected biomass enzymatic saccharification and plant lodging resistance in the *AtCESA8::SUS3* transgenic rice plants. Furthermore, we detected any alterations of cell wall compositions and wall polymers features in the transgenic plants, and proposed a model that shows how *OsSUS3* overexpression leads to largely improved biomass enzymatic saccharification and plant lodging resistance by altering cellulose crystallinity and cell wall thickness.

## Results

### *OsSUS3*-transgenic plants maintain a normal growth with slightly increased biomass yields

**Fig. 1:**
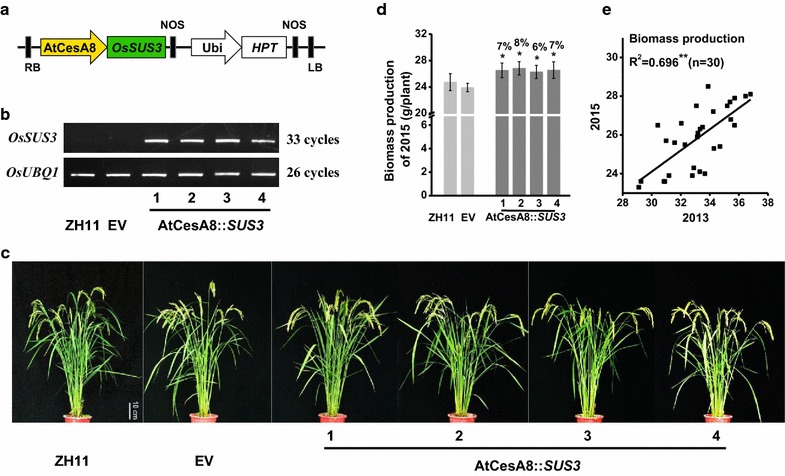
Selection of AtCESA8::*SUS3* transgenic plants. **a** Gene construct used to generate transgenic rice plants. **b** RT-PCR analysis of *OsSUS3* in the transgenic lines. **c** Phenotype observation of the transgenic rice plants at filling stage (scale bar = 10 cm). **d** Mature stem biomass yield per plant harvested in the year of 2015. All data are given as mean ± SD. A Student’s *t* test was performed between the transgenic plants and ZH11 as **P < 0.01 and *P < 0.05 (*n* = 3). **e** Correlation analysis of biomass yields between 2013 and 2015

Based on the public microarray database, we initially observed a low expression of *OsSUS3* gene in the tissues that are rich at secondary cell walls in rice (Additional file 1: Figure S1). To enhance *OsSUS3* expression, we selected the transgenic rice plants that expressed *OsSUS3* driven by AtCesA8 promoter (Fig. 1a), which mainly drives cellulose synthase (CESA) gene’s expression in the secondary cell wall synthesis of *Arabidopsis* [26]. As a result, the selected four independent homozygous lines were examined with much higher *OsSUS3* transcript levels in the stem tissues of transgenic rice plants, compared with the controls including ‘Zhonghua11′ (ZH11) cultivar and the transgenic line-expressed empty vector (EV) (Fig. 1b), which was confirmed by Western analysis (data not shown).

During the 2-year field experiments, all transgenic rice lines showed a normal growth and development over the life cycles of rice, compared with the ZH11 and EV line (Fig. 1c). However, the transgenic rice plants achieved slightly increased biomass yields by 6–8% at *P* < 0.05 and 0.01 levels (Fig. 1d; Additional file 1: Table S1), and the biomass yields were even consistent in two-year field experiments, based on a significantly positive correlation (Fig. 1e). The results suggest that overexpression of *OsSUS3* has little effect on plant growth with the slightly increased biomass yields in transgenic rice plants.

### Enhanced biomass saccharification and bioethanol production in the *OsSUS3*-transgenic lines

**Fig. 2:**
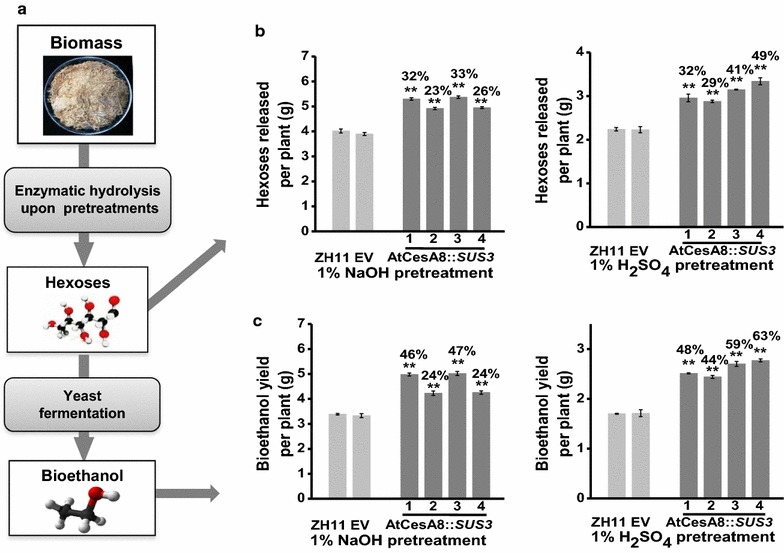
Biomass enzymatic saccharification and ethanol production of the *OsSUS3*-transgenic rice plants. **a** The schematic flow for biomass saccharification and ethanol production. **b** Hexose yields released from enzymatic hydrolysis after the pretreatment with 1% NaOH or 1% H_2_SO_4_. **c** Bioethanol yields obtained from yeast fermentation using the sugars released from biomass enzymatic hydrolysis as performed in **b**. All data are given as mean ± SD. A Student’s *t* test was performed between transgenic plants and ZH11 as **P < 0.01 and *P < 0.05 (*n* = 3)

In this study, biomass enzymatic saccharification (digestibility) was assessed by calculating the hexose yields released from enzymatic hydrolysis after chemical pretreatments of biomass residues in the mature stem tissues of rice (Fig. 2a). In general, all the four AtCesA8::*SUS3* transgenic lines exhibited significantly higher biomass saccharification than those of ZH11 and EV at *P* < 0.01 levels, with the hexoses yields being increased by 23–33 and 29–49% (per plant) or by 15–23 and 21–39% (per dry matter) released from enzymatic hydrolysis after 1% NaOH and 1% H_2_SO_4_ pretreatments, respectively (Fig. 2b; Additional file 1: Figure S2a). Consequently, the AtCesA8::*SUS3* transgenic plants had the bioethanol yields increased by 24–47 and 44–63% (per plant) or by 13–37 and 29–54% (per dry matter), respectively, obtained from yeast fermentation (Fig. 2c; Additional file 1: Figure S2b). Hence, the overexpression of *OsSUS3* led to largely enhanced biomass saccharification and bioethanol production in rice.

### Increased lodging resistance and mechanical strength in the *OsSUS3*-transgenic lines

Three criteria have been defined for evaluating plant lodging resistance. In particular, lodging index (LI) is highly related to plant height, fresh weight, and breaking force, which was the direct criterion that negatively accounts for plant lodging resistance [21, 36]. Extension and pushing forces are the other criteria accounting for the elasticity and mechanical strength of plant organs and tissues [8, 36–39]. In this study, we detected lodging index of the transgenic lines and controls from the two-year field experiments. All the four independent transgenic lines exhibited reduced lodging index values by 17–50%, compared to those in ZH11 and EV. Meanwhile, we examined significantly increased extension force by 8–17% and pushing force by 13–44% in the AtCesA8::*SUS3* transgenic lines (Table 1). Because plant extension and pushing forces are tightly associated with lodging resistance [8, 36–39], all data were thus consistent with the findings about the significantly increased lodging resistance in the transgenic rice plants.

**Table 1:** Detection of lodging index, extension, and pushing forces of the *OsSUS3*-transgenic lines in field experiments

Transgenic lines	Lodging index				Extension force (*N*)				Pushing force (*N*)	
	2013		2015		2013		2015		2015	
Vector										
ZH11	170.62 ± 7.66		269.21 ± 8.47		173.80 ± 8.92		202.93 ± 7.18		1.66 ± 0.13	
EV	166.70 ± 6.34		278.64 ± 16.1		178.96 ± 4.28		206.78 ± 6.97		1.69 ± 0.14	
AtCesA8::SUS3										
1	108.14 ± 6.13**	−37%^a^	221.18 ± 7.59**	−18%	190.78 ± 3.08*	+10%	230.42 ± 4.79**	+14%	2.31 ± 0.10**	+39%
2	108.96 ± 7.82**	−36%	188.84 ± 22.72**	−30%	202.67 ± 15.59**	+17%	222.23 ± 3.54**	+10%	2.01 ± 0.06**	+21%
3	97.72 ± 8.57**	−43%	211.07 ± 11.23**	−22%	193.80 ± 11.04*	+12%	219.32 ± 3.67**	+8%	1.88 ± 0.09**	+13%
4	85.62 ± 9.24**	−50%	223.44 ± 14.06**	−17%	196.26 ± 11.28**	+13%	224.85 ± 12.46**	+11%	2.39 ± 0.10**	+44%

* and **, indicated significant difference between transgenic lines and ZH11 control by *t* test as *P* < 0.05 and 0.01 (*n* = 10)

^a^Percentage of increased or decreased level between transgenic line and ZH11 by subtraction of two values divided by ZH11

### Altered cell wall ultrastructure and composition in the *OsSUS3*-transgenic lines

**Fig. 3:**
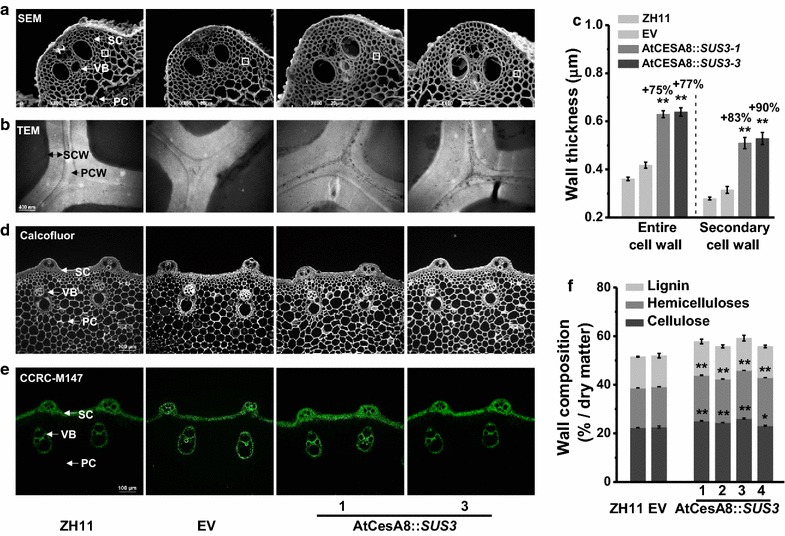
Observations of plant cell wall structures in the *OsSUS3*-transgenic rice plants. **a** Scanning electron microscopy (SEM) images of the second internode stem at the heading stage of rice: sclerenchyma cells (SC); vascular bundle cells (VB); and parenchyma cells (PC) (scale bars as 20 μm). **b** Transmission electron microscopy (TEM) images of the sclerenchyma cell walls (*PCW* primary cell wall, *SCW* secondary cell wall; scale bars = 400 nm). **c** Quantitative measurement of cell wall thickness by TEM in **b** (20 cells). **d** Calcofluor (white) staining specific for cellulose (scale bars as 100 μm). **e** Immunohistochemical staining (green) specific for xylan, using CCRC-M147 antibody (scale bars as 100 μm). **f** Cell wall compositions of mature stems including cellulose, hemicelluloses, and lignin. All data are given as mean ± SD; Student’s *t* test between ZH11 and transgenic plants as **P < 0.01 and *P < 0.05 (*n* = 3)

To understand enhanced biomass digestibility and lodging resistance of the *OsSUS3*-transgenic plants, we examined cell wall ultrastructure using scanning electron microscopy and transmission electron microscopy (Fig. 3). As a result, two representative AtCesA8::*SUS3* transgenic lines obviously exhibited thickened sclerenchyma cells (SC), vascular bundle cells (VB), and parenchyma cells (PC), compared to those in ZH11 and EV (Fig. 3a, b). In particular, the widths of entire cell walls and secondary cell walls in the sclerenchyma cells were respectively increased by 75–77 and 83–90% in the AtCesA8::*SUS3* transgenic plants, compared with the controls (Fig. 3c). Hence, the overexpression of *OsSUS3* led to remarkably increased secondary cell wall thickness in the transgenic rice plants.

Furthermore, we measured cell wall compositions of the AtCesA8::*SUS3* transgenic plants including cellulose, hemicelluloses, and lignin. As a comparison, the four transgenic lines had significantly increased cellulose levels at *P* < 0.05 and 0.01 levels (Fig. 3f; Additional file 1: Table S2), consistent with the Calcofluor staining specific for cellulose in the stem tissues (Fig. 3d). Upon using the plant glycan-directed monoclonal antibody (CCRC-M147), we observed that the AtCesA8::*SUS3* transgenic plants showed much brighter fluorescent signals specific for unsubstituted xylan of hemicelluloses in the thickened sclerenchyma cells and vascular bundle cells (Fig. 3e), consistent with the significantly increased hemicelluloses levels by 9–21% in the transgenic lines (Fig. 3f; Additional file 1: Table S2). Meanwhile, we demonstrated that the four AtCesA8::*SUS3* transgenic lines had lignin levels similar to those of ZH11 and EV in the mature stems (Fig. 3f; Additional file 1: Table S2). Taking all these findings together, it is concluded that the overexpression of *OsSUS3* led to enhanced cellulose and hemicelluloses depositions into the cell walls, consistent with the findings of the more-thickened cell walls and increased biomass yields in transgenic rice plants.’

### Reduced cellulose crystallinity for enhanced biomass saccharification and lodging resistance

**Fig. 4:**
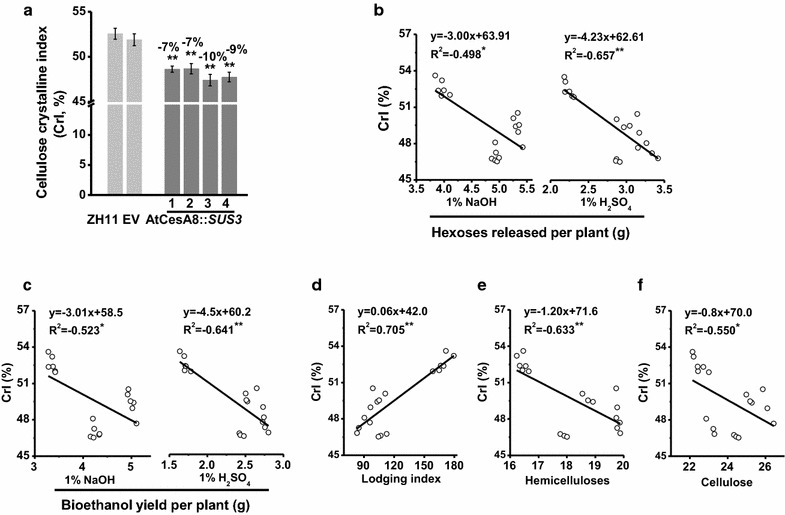
Characterization of cellulose crystallinity in the *OsSUS3*-transgenic rice plants. **a** Measurement of cellulose crystallinity index (CrI, %). Correlation between cellulose CrI values and hexoses yields (**b**) or bioethanol yields (**c**) or lodging index (**d**) or hemicelluloses levels (**e**) or cellulose levels (**f**). * and ** indicated significant correlations at *P* < 0.05 and 0.01 (*n* = 18), respectively

Cellulose crystallinity has been well characterized as the key negative factor on biomass enzymatic digestibility in different plant species [5, 19, 20]. Using X-ray detection method, this study measured cellulose crystalline index (CrI) of the AtCesA8::*SUS3* transgenic plants, which is accounting for cellulose crystallinity [40–42]. As a comparison, all four transgenic rice lines had much lower CrI values by 7–10% than those of ZH11 and EV in the mature stem tissues (Fig. 4a). Because correlation analysis has been well applied to account for wall polymer feature impacts on biomass enzymatic saccharification in different plant species [5, 19, 20], we performed a correlation analysis between cellulose CrI and biomass saccharification or bioethanol production in all transgenic lines and controls. As a result, cellulose CrI values were negatively correlated with either the hexoses yields released from enzymatic hydrolysis after chemical pretreatments or the bioethanol yields from yeast fermentations at *P* < 0.05 or 0.01 levels, with high *R* ^2^ values at 0.498–0.657 (Fig. 4b, c). These data thus suggest that the reduction of cellulose CrI should be a major influential factor on the enhanced biomass saccharification and bioethanol production in the AtCesA8::*SUS3* transgenic plants, consistent with previous reports in other biomass samples [19, 40, 41]. Notably, the cellulose CrI also showed a significantly positive correlation with lodging index at *P* < 0.01 levels, with high *R* ^2^ values at 0.705 (Fig. 4d), indicating that the reduced cellulose CrI should also be a major influential factor on the increased lodging resistance in transgenic rice plants. In addition, total hemicelluloses and cellulose levels exhibited a negative correlation with cellulose CrI values at *P* < 0.01 and 0.05 levels (Fig. 4e, f), consistent with the previous reports of hemicelluloses and cellulose having negative impacts on cellulose crystallinity [19].

### Altered gene expressions involved in wall polymer synthesis

**Fig. 5:**
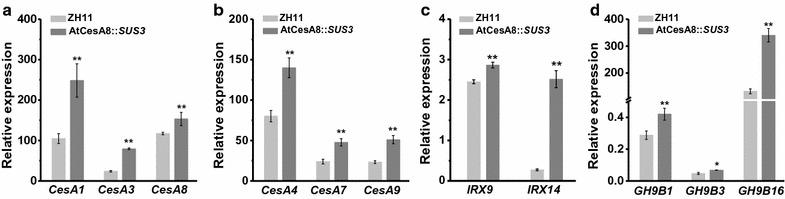
qRT-PCR analysis of gene expressions associated with wall biosynthesis and modification in the *OsSUS3*-transgenic rice plants. **a** Genes involved in primary wall cellulose biosynthesis. **b** Genes involved in secondary wall cellulose biosynthesis. **c** Genes associated with Xylan backbone synthesis. **d** Genes associated with cellulose modifications. * and ** indicated significant different transcription levels between the transgenic rice and ZH11 control by *t* test at *P* < 0.05 and 0.01 (*n* = 3), respectively

Because cellulose and hemicelluloses contents are increased in the AtCesA8::*SUS3* transgenic rice plants, we examined transcript levels of the genes that are involved in two wall polymers syntheses. As OsCesA1, 3, 8 and OsCesA4, 7, 9 are, respectively, involved in the cellulose synthesis of the primary and secondary cell walls in rice [42, 43], we demonstrated that those six *OsCesAs* genes had much higher expressions in the representative AtCesA8::*SUS3* transgenic plants at *P* < 0.01 level, compared to their expressions in the ZH11 control (Fig. 5a, b), which is consistent with the increased cellulose levels in the transgenic plants. The AtCesA8::*SUS3* transgenic plant also showed much higher transcript levels of *OsIRX9* and *OsIRX14* than those of the ZH11 control at *P* < 0.01 levels (Fig. 5c). Because *OsIRX9* and *OsIRX14* have been identified to catalyze xylan backbone synthesis in rice [44, 45], the results were consistent with the increased hemicelluloses levels in the AtCesA8::*SUS3* transgenic plants.

Furthermore, we examined transcript levels of three representative *OsGH9B* genes in the AtCesA8::*SUS3* transgenic plants, because those three enzymes may have activity for reducing cellulose crystallinity in rice [19, 46]. Significantly, the AtCesA8::*SUS3* transgenic plant exhibited much higher *OsGH9B* expression levels than those of the ZH11 control (Fig. 5d), consistent with the findings of reduced cellulose CrI in the transgenic rice plants.

## Discussion

Plant cell walls represent the most abundant renewable biomass resources for bioethanol production [1, 47] and largely affect mechanical strength of plants [21, 39, 48]. It has been characterized that plant cell wall compositions and wall polymer features play distinctive roles in biomass recalcitrance and plant lodging resistance [12–21]. Although genetic modification of plant cell walls has been posed as a promising solution to the recalcitrance and lodging [22–25], it remains a challenge to maintain normal growth and mechanical strength in transgenic plants. It thus becomes crucial to identify critical genes and appropriate promoters for selection of transgenic plants that are of improved biomass saccharification and lodging resistance [47]. In this study, the AtCesA8::*SUS3* transgenic rice plants could not only maintain a normal growth with slightly increased biomass yield, but also exhibited significantly enhanced biomass saccharification and lodging resistance, indicating a powerful genetic strategy for cell wall modification in bioenergy crops. Although *SUS* transgenic plants have previously shown improvements in cellulose and starch contents in poplar, cotton, and potato [27–35], this study for the first time demonstrated the enhancement role of *SUS* in plant lodging resistance, which is one of the most important agronomic traits tightly associated with grain and biomass yields in crops.

**Fig. 6:**
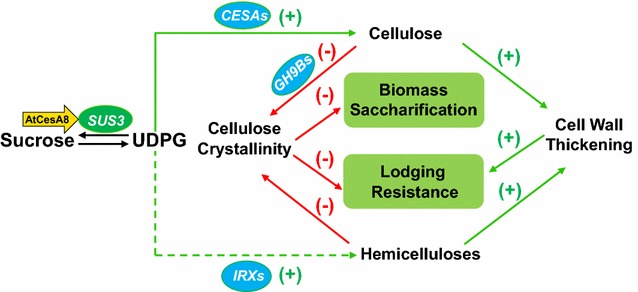
A hypothetical model that highlights how OsSUS3 positively regulates cellulose and hemicelluloses biosynthesis to reduce cellulose crystallinity and to increase cell wall thickness for enhancing both biomass saccharification and lodging resistance in the transgenic rice plants. (−) and (+) indicated as negative and positive factors (or impacts), respectively

To understand why the AtCesA8::*SUS3* transgenic rice plants are of improved saccharification and lodging resistance, we propose a mechanistic model to elucidate that the enhanced OsSUS3 activity could provide sufficient UDPG substrate for direct cellulose biosynthesis and indirect hemicelluloses production, leading to reduced cellulose crystallinity and increased cell wall thickness in the transgenic rice plants (Fig. 6). The model hence highlights two major wall factors (cellulose crystallinity and cell wall thickening) that distinctively affect biomass enzymatic saccharification and plant lodging resistance in the transgenic rice plants. The cellulose crystallinity could negatively affect both biomass saccharification and plant lodging resistance, whereas the cell wall thickening mainly increase lodging resistance. Furthermore, it assumes that cellulose crystallinity should be negatively affected by both the cellulose modification with GH9B enzymes and the hemicelluloses deposition into the transgenic rice plants. The model also indicates that the syntheses of increased wall polymers (cellulose and hemicelluloses) with CESAs and IRXs enzymes should be the major factors of influence for the cell wall thickening in the transgenic plants.

It has been characterized that the cellulose CrI values reflect the relative amounts of crystalline materials and that the cellulose microfibrils with high crystallinity is thus less accessible by cellulases in biomass enzymatic hydrolysis [49]. On the other hand, the reduced cellulose CrI may reflect an increased amorphous density of cellulose microfibrils that allows for more cellulase enzyme loading and facile accessibility, and this study has thus demonstrated that cellulose crystallinity is the key factor that negatively affects biomass enzymatic digestibility and bioethanol production in the AtCESA8::*SUS3* transgenic plants. In terms of the reduced cellulose crystallinity in the AtCESA8::*SUS3* transgenic plants, this study has provided two evidences: increased expressions of three representative OsGH9B genes and an increased hemicelluloses level, which has previously been reported to negatively affect cellulose crystallinity in rice and other plants [19, 46, 50, 51]. However, it remains to analyze cell wall and cellulose ultrastructures in the AtCESA8::*SUS3* transgenic plants in the future, which may provide a direct evidence about the reduced cellulose crystallinity.

As plant cell walls basically determine plants’ mechanical strength [12–19, 52], the increased cell wall thickness should be a major influential factor for the enhanced lodging resistance in the transgenic rice plants. Meanwhile, the increased extension and pushing forces should be also due to the cell wall thickenings in the transgenic plants, which is another evidence for the enhanced lodging resistance [36]. Because cellulose crystallinity negatively affects lodging index in rice, the reduction of cellulose crystallinity should be an additional contributor to the lodging resistance in the transgenic plants.

In addition, although the *OsSUS3*-transgenic plants had slightly increased biomass yield, they may have largely enhanced biomass production if grown in the large fields fed with good amounts of fertilizers in the future, because this study has witnessed a very high increase in cellulose and hemicelluloses levels and cell wall thickness. The previous report has indicated that the SUS transgenic tree plants have a much enhanced biomass productivity [32, 33]. Therefore, the enhancement of cellulose accumulation in raw biomass and improvement of biomass digestibility will potentially reduce the costs of lignocellulosic biofuel productions [53]. In addition, those four independent SUS3 transgenic lines displayed significantly decreased sucrose levels (Additional file 1: Table S3), suggesting that the improvements in the trait of transgenic plants are most likely due to an efficient energy allocation and consumption, particularly for cellulose synthesis.

## Conclusions

Overexpression of *OsSUS3* driven by *AtCESA8* promoter could not only lead to enhanced biomass saccharification and bioethanol production after chemical pretreatments, but it also caused improved lodging resistance in the transgenic rice plants. Furthermore, this study has indicated that the reduction of cellulose crystallinity is a major factor for enhancements of both biomass saccharification and lodging resistance, and the increase of wall thickness may be the additional contributor to mechanical strength and lodging resistance in the transgenic rice plants. Hence, the results provide new insights into sucrose synthase regulation on carbon partitioning for cellulose and other polysaccharide biosynthesis. It also provides a powerful genetic approach for cell wall modification in bioenergy crops.

## Methods

### Vector constructs and transformation

The full-length cDNA of *SUS3* was cloned from rice cultivar ‘Nipponbare’ (a *japonica* variety) and sequenced. It was inserted into the plant binary vector pCAMBIA1300 (Cambia) under the *Arabidopsis* promoter AtCESA8. The constructs were introduced into *Agrobacterium tumefaciens* strain EHA105 and transferred to rice cultivar ‘Zhonghua11’ (ZH11) by *Agrobacterium*-mediated transformation. Ten sibling transgenic plants were assayed at each of the T_2_–T_4_ generations. All primers used for gene cloning are listed in Additional file 1: Table S4.

### Total RNA isolation and RT-PCR analysis

Samples were collected from stems at heading stage. Total RNAs were extracted using Trizol reagent (Invitrogen, Carlsbad, CA, USA) and reverse-transcribed into cDNA with the GoScript™ Reverse Transcription System (Promega, USA). The RT-PCR reaction was performed as described previously [43]. Quantitative real time-PCR (qRT-PCR) was independently performed in triplicate using the SYBR Green PCR Master Mixture (ZF101, ZOMANBIO). A rice *polyubiquitin* gene (*OsUBQ1*) was used as the internal control. All primers used for RT-PCR are listed in Additional file 1: Table S4.

### Field experiments and trait measurement

Transgenic rice plants were grown under natural field conditions in the experimental stations of Huazhong Agricultural University, Wuhan, China. Conventional rice cropping practices, including irrigation, fertilizer application, and pest control, were applied to the field experiments in this study.

### Plant lodging index and mechanical property measurement

Plant lodging index (LI), extension force (EF) and pushing force (PF) were measured as described previously [19, 21, 36, 39]. All experiments were conducted on duplicate samples of ten sibling transgenic plants at each line, using the stem tissues at 30 days after flowering. The breaking resistance of the third internode was detected using a Prostrate Tester (DIK 7401, Daiki Rika Kogyo Co. Ltd., Tokyo, Japan), with the distance between fulcra of the tester at 5 cm. Fresh weight (W) of the upper portion of the plant was measured including panicle and the three internodes, leaf and leaf sheath. Bending moment (BM) and lodging index (LI) were calculated using the following formula: BM = Length from the third internode to the top of panicle × W, LI = BM/breaking resistance. The EF were the stretching force of the samples before being broken, measuring with a universal force/length testing device (model RH-K300, Guangzhou, China). The PF were measured with five stems in each experimental unit (plot) by the prostrate tester (DIK-7400, Japan). The newton is used as the unit of EF and PF.

### Plant cell wall fractionation and determination

The plant tissues were dried to constant weight, ground using a knife-mill and passed through a 40-mesh screen. Plant cell wall fractionation method and total cellulose and hemicelluloses assay were conducted as described previously [19, 21]. For crystalline cellulose extraction, biomass samples (0.1 g) were added with 5.0 mL acetic acid–nitric acid–water (8:1:2, v/v/v) and heated for 1 h in a boiling water bath with stirring every 10 min. After centrifugation, the pellet was washed several times with 5.0 mL water and dissolved in 67% H_2_SO_4_. Total hexoses in 67% H_2_SO_4_ were regarded as cellulose. For hemicelluloses analysis, the dry biomass powder samples (0.1 g) were treated by potassium phosphate buffer (pH 7.0), chloroform–methanol (1:1, v/v), DMSO–water (9:1, v/v), and 0.5% (w/v) ammonium oxalate to remove soluble sugar, lipids, starch, and pectin. The remaining pellet was suspended in 4 M KOH containing 1.0 mg mL^−1^ sodium borohydride for 1 h at 25 °C, and the combined supernatant was neutralized, dialyzed and lyophilized for total hemicelluloses analysis.

Total lignin content includes acid-insoluble and -soluble lignin was determined by two-step acid hydrolysis method as previously described [54]. The sample (0.5 g, *W*1) was extracted with benzene–ethanol in a Soxhlet for 4 h, and then air dried. The sample was hydrolyzed with 10 mL 72% H_2_SO_4_ (v/v) in shaker at 30 °C for 1.5 h. After hydrolysis, the acid was diluted to a concentration of 2.88%, and then placed in the autoclave for 1 h at 121 °C. The autoclaved hydrolysis solution was vacuum-filtered through the filtering crucible. The filtrate was captured in a filtering flask for acid-soluble lignin. The acid-soluble lignin was solubilized during the hydrolysis process, and was measured by UV spectroscopy. The acid-insoluble residue was washed free of acid with distilled water and dried in an oven until attaining a constant weight. The weights of the crucible and dry residue were recorded (*W*2). Finally, the dried residues were ashed in the muffle furnace at 200 °C for 30 min and 575 °C for 4 h. The crucibles and ash were weighed, and their weights were recorded (*W*3). Acid-insoluble lignin (AIL) on original sample was calculated as follows: AIL (%) = (*W*2 − *W*3) × 100/*W*1%. Total lignin (%) = ASL% + AIL%. All experiments were conducted using biological triplicates.

### Cellulose CrI detection

The X-ray diffraction (XRD) method was applied for detection of the lignocellulose crystalline index (CrI) in the crude cell wall materials using Rigaku-D/MAX instrument (Ultima III; Japan) as described by [46, 55–57]. The raw biomass powder was laid on the glass sample holder (35 × 50 × 5 mm) and detected under plateau conditions. Ni-filtered Cu-Kα radiation (*λ* = 0.154056 nm) was generated at voltage of 40 kV and current of 18 mA, and scanned at speed of 0.0197°/s from 10 to 45°. The CrI was estimated using the intensity of the 200 peak (*I*_200_, *θ* = 22.5°) and the intensity at the minimum between the 200 and 110 peaks (*I*_am_, *θ* = 18.5°) as follows: CrI = 100 × (*I*_200_ − *I*_am_)/*I*_200_. The XRD method was detected with representative samples in triplicate.

### Biomass pretreatment and enzymatic hydrolysis

The chemical (NaOH, H_2_SO_4_) pretreatment and sequential enzymatic hydrolysis were performed as described previously with minor modifications [57]. For NaOH pretreatment, the ground biomass powder was supplemented with 6 mL 1% NaOH (w/v). For H_2_SO_4_ pretreatment, the biomass powder was added with 6 mL 1% H_2_SO_4_ (v/v) and heated at 121 °C for 20 min in autoclave. The samples of chemical (NaOH, H_2_SO_4_) pretreatments were shaken at 150 r/min for 2 h at 50 °C, and centrifuged at 3000*g* for 5 min. The remaining pellet was washed three times with 10 mL distilled water, and all supernatants were combined for sugar analysis. The remaining residue was collected for enzymatic hydrolysis. Samples with 6 mL distilled water only were shaken for 2 h at 50 °C as control. All experiments were performed in the biological triplicates.

### Yeast fermentation and ethanol measurement

Yeast fermentation and ethanol measurement were performed as previously described by Jin et al. [58] with minor modification. After pretreatments, the biomass residues and supernatants were neutralized to pH 4.8 using appropriate amounts of H_2_SO_4_ or NaOH. Then, mixed-cellulases were added to the final enzyme concentration at 1.6 g/L, and incubated at 150 r/min for 48 h at 50 °C. *Saccharomyces cerevisiae* (Angel yeast Co., Ltd., Yichang, China) was used in all fermentation reactions, and the yeast powder was dissolved in 0.2 M phosphate buffer (pH 4.8) for 30 min for activation prior to use. The activated yeast (ferment hexoses only) was inoculated into the mixture of enzymatic hydrolysates and residues with initial cell mass concentration at 0.5 g/L. The fermentation experiments were performed at 37 °C for 48 h, and distilled for determination of ethanol content. Ethanol content was measured using the dichromate oxidation method. All experiments were performed in the biological triplicates.

### Scanning electron microscopy and transmission electron microscopy analyses

The second internodes (0.5 cm sections above the node) at the heading stage were cut into 1–2 mm pieces, subsequently fixed with 2.5% (v/v) glutaraldehyde, vacuumed three times, and fixed for at least 24 h. Samples were natural dried, sputter-coated with gold particles, observed, and photographed using a scanning electron microscope (JSM-6390LV; JEOL, Japan). Scanning electron microscopy (SEM) analysis was based on at least three biological replications of the mounted specimens. All procedures were carried out according to the manufacturer’s protocol.

Transmission electron microscopy (TEM) was used to observe cell wall structures in the middle 0.5 cm sections from the third leaf veins of three-leaves-old plants. Tissues were high-pressure frozen, freeze substituted, embedded, sectioned, and viewed according to McFarlane et al. [59]. The samples were post-fixed in 2% (w/v) OsO_4_ for 1 h after extensively washing in the PBS buffer and embedded with Suprr Kit (Sigma). Sample sections were cut with an Ultracut E ultrami-crotome (Leica) and picked up on formvar-coated copper grids. After post-staining with uranyl acetate and lead citrate, the specimen were viewed under a Hitachi H7650 (Hitachi Ltd., Tokyo, Japan) transmission electron microscope. The width of cell wall was measured using the software ImageJ (NIH, USA), and more than 40 cell walls each for the different genotypes were measured. Significance was estimated using Student’s *t* test.

### Microscope observation

The sample preparation was performed as previously described [60]. The second internodes (0.5 cm sections above the node) at the heading stage were cut into pieces, subsequently fixed with 4% (w/v) paraformaldehyde, and dehydrated through an ethanol gradient (30, 50, 70, 90, 100 and 100%, each for 30 min), and then embedded in paraplast plus. The sections (8 µm thickness) were cut using a microtome (RM2265, Leica) and placed on lysine-treated slides which were dried for 2 days at 37 °C, and de-waxed with xylene and hydrated through an ethanol series (100–0%). The sections were treated with PBS buffer contained 3% SMP (skim milk powder, w/v) for 1 h, and incubated with PBS containing 10 µg/mL CCRC-147 (http://glycomics.ccrc.uga.edu/wall2/antibodies/antibodyHome.html, CCRC-M147 recognized selectively all structures containing an unsubstituted xylan disaccharide) [61] for another 1 h. The immunolabeled samples were washed three times (5 min each) with PBS and incubated with a 100-fold dilution of anti-mouse-IgG in dark for 2 h. The anti-mouse-IgG antibody was labeled by fluorescein-isothiocyanate (FITC). Counterstaining was performed with Calcofluor white M2R fluorochrome (fluorescent brightener 28; Sigma; 0.25 μg/mL in dH_2_O). Immunofluorescence sections were imaged using a microscope (Olympus BX-61, Japan) equipped with the following filter sets: 350/450 nm (ex/em) for visualizing calcofluor white stained cell walls, and 490/520 nm (ex/em) for green emission of the FITC fluorochrome, respectively.

### Statistical calculation of correlation coefficients

Both two-tailed Student’s *t* test and analysis of variance (ANOVA) were performed using SPSS. Significance was measured at the levels of *P* < 0.05 and *P* < 0.01. Correlation coefficients were calculated by performing Spearman’s rank correlation analysis for all pairs of measured traits across the whole population.

## Additional file

**Additional file 1: Figure S1.** Gene expression profiling of *OsSUS3* in life cycle of rice. **Figure S2.** Biomass enzymatic saccharification and ethanol production of the *OsSUS3*-transgenic rice plants. (a) Hexose yields released from enzymatic hydrolysis after the pretreatment with 1% NaOH or 1% H_2_SO_4_. (b) Bioethanol yields obtained from yeast fermentation using the sugars released from biomass enzymatic hydrolysis as performed in (a). All data are given as means ± SD. A Student’s *t*-test was performed between transgenic plants and ZH11 as ***P* < 0.01 and **P* < 0.05 (n = 3).

## Abbreviations

SUS: sucrose synthase
UDPG: UDP-glucose
CrI: cellulose crystallinity index
LI: lodging index
EF: extension force
PF: pushing force
SEM: scanning electron microscopy
TEM: transmission electron microscopy
SC: sclerenchyma cells
VB: vascular bundle cells
PC: parenchyma cells
CESA: cellulose synthase
GH: glycoside hydrolase
