# Supplementary material for: AtCesA8-driven OsSUS3 expression leads to largely enhanced biomass saccharification and lodging resistance by distinctively altering lignocellulose features in rice
Source: Biotechnol Biofuels. 2017 Sep 16;10:221. doi: 10.1186/s13068-017-0911-0 (PMC5603028; doi:10.1186/s13068-017-0911-0)
Supplement: Supplementary file 1 — Additional file 1: Figure S1. Gene expression profiling of OsSUS3 in life cycle of rice. Figure S2. Biomass enzymatic saccharification and ethanol production of the OsSUS3-transgenic rice plants. (a) Hexose yields released from enzymatic hydrolysis after the pretreatment with 1% NaOH or 1% H2SO4. (b) Bioethanol yields obtained from yeast fermentation using the sugars released from biomass enzymatic hydrolysis as performed in (a). All data are given as means ± SD. A Student’s t-test was performed between transgenic plants and ZH11 as **P < 0.01 and *P < 0.05 (n = 3). [file 13068_2017_911_MOESM1_ESM.ppt]

## Slide 1
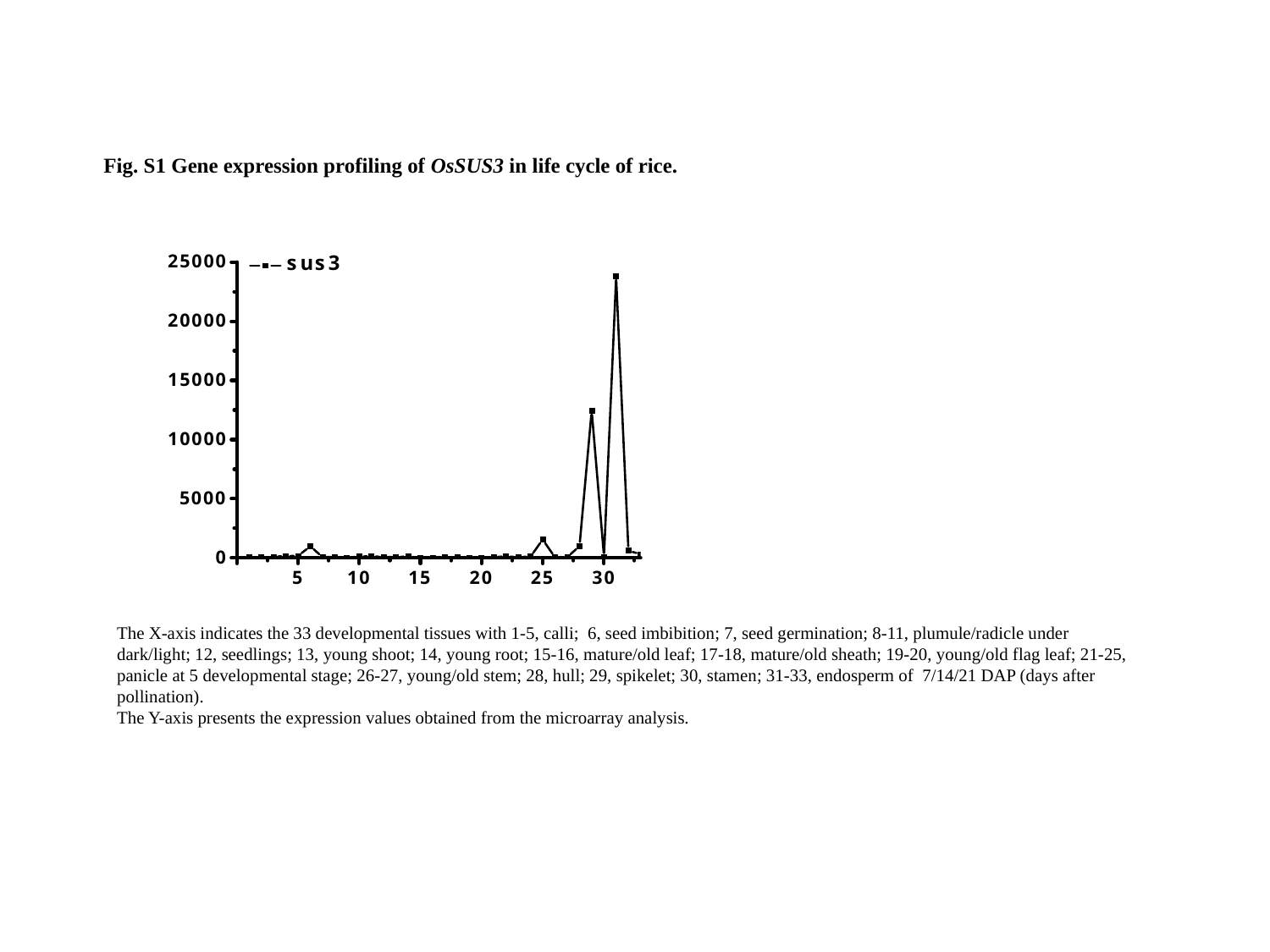

# Fig. S1 Gene expression profiling of OsSUS3 in life cycle of rice.
The X-axis indicates the 33 developmental tissues with 1-5, calli; 6, seed imbibition; 7, seed germination; 8-11, plumule/radicle under dark/light; 12, seedlings; 13, young shoot; 14, young root; 15-16, mature/old leaf; 17-18, mature/old sheath; 19-20, young/old flag leaf; 21-25, panicle at 5 developmental stage; 26-27, young/old stem; 28, hull; 29, spikelet; 30, stamen; 31-33, endosperm of 7/14/21 DAP (days after pollination).
The Y-axis presents the expression values obtained from the microarray analysis.

## Slide 2
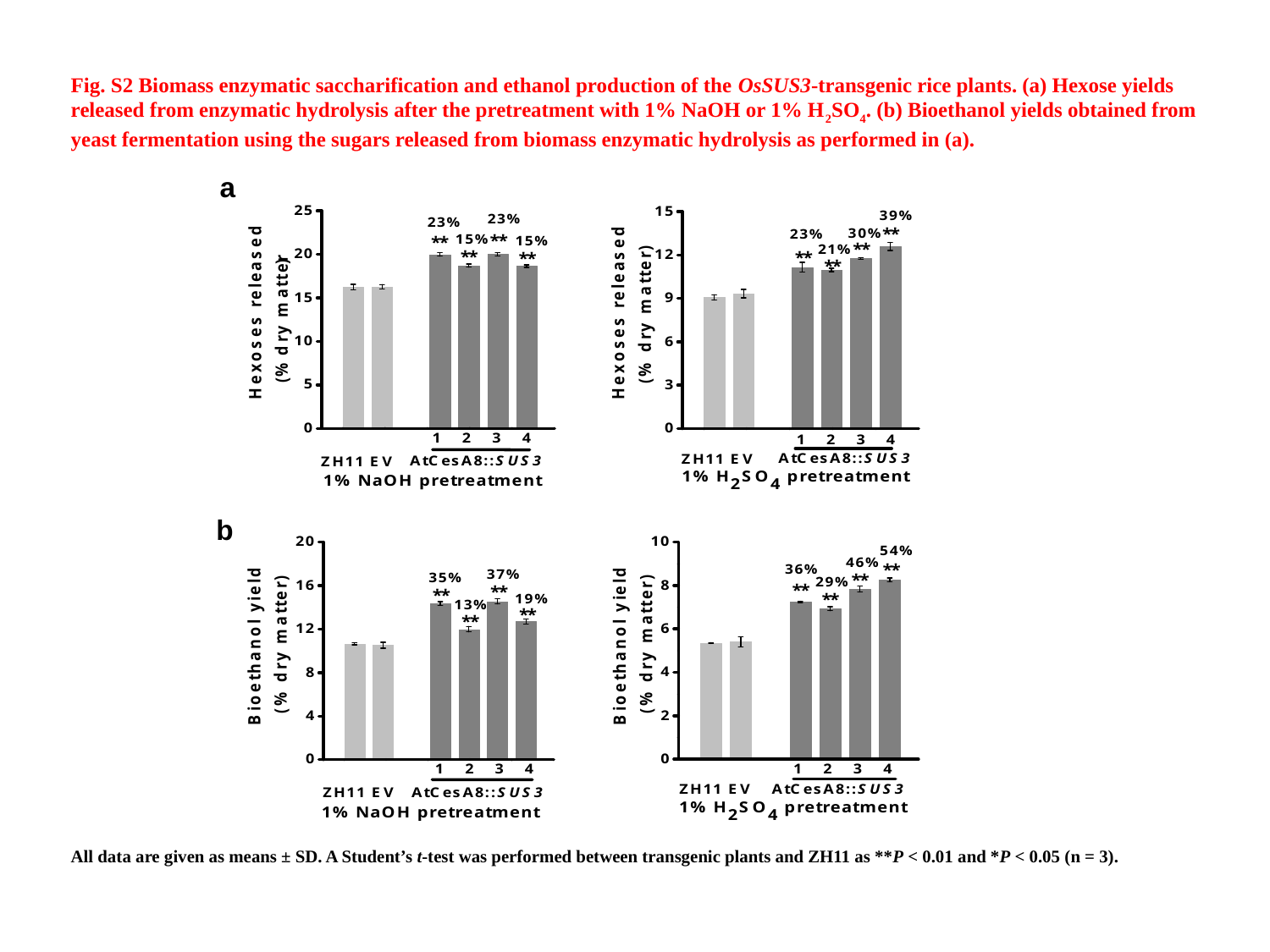

Fig. S2 Biomass enzymatic saccharification and ethanol production of the OsSUS3-transgenic rice plants. (a) Hexose yields released from enzymatic hydrolysis after the pretreatment with 1% NaOH or 1% H2SO4. (b) Bioethanol yields obtained from yeast fermentation using the sugars released from biomass enzymatic hydrolysis as performed in (a).
a
b
All data are given as means ± SD. A Student’s t-test was performed between transgenic plants and ZH11 as **P < 0.01 and *P < 0.05 (n = 3).

## Slide 3
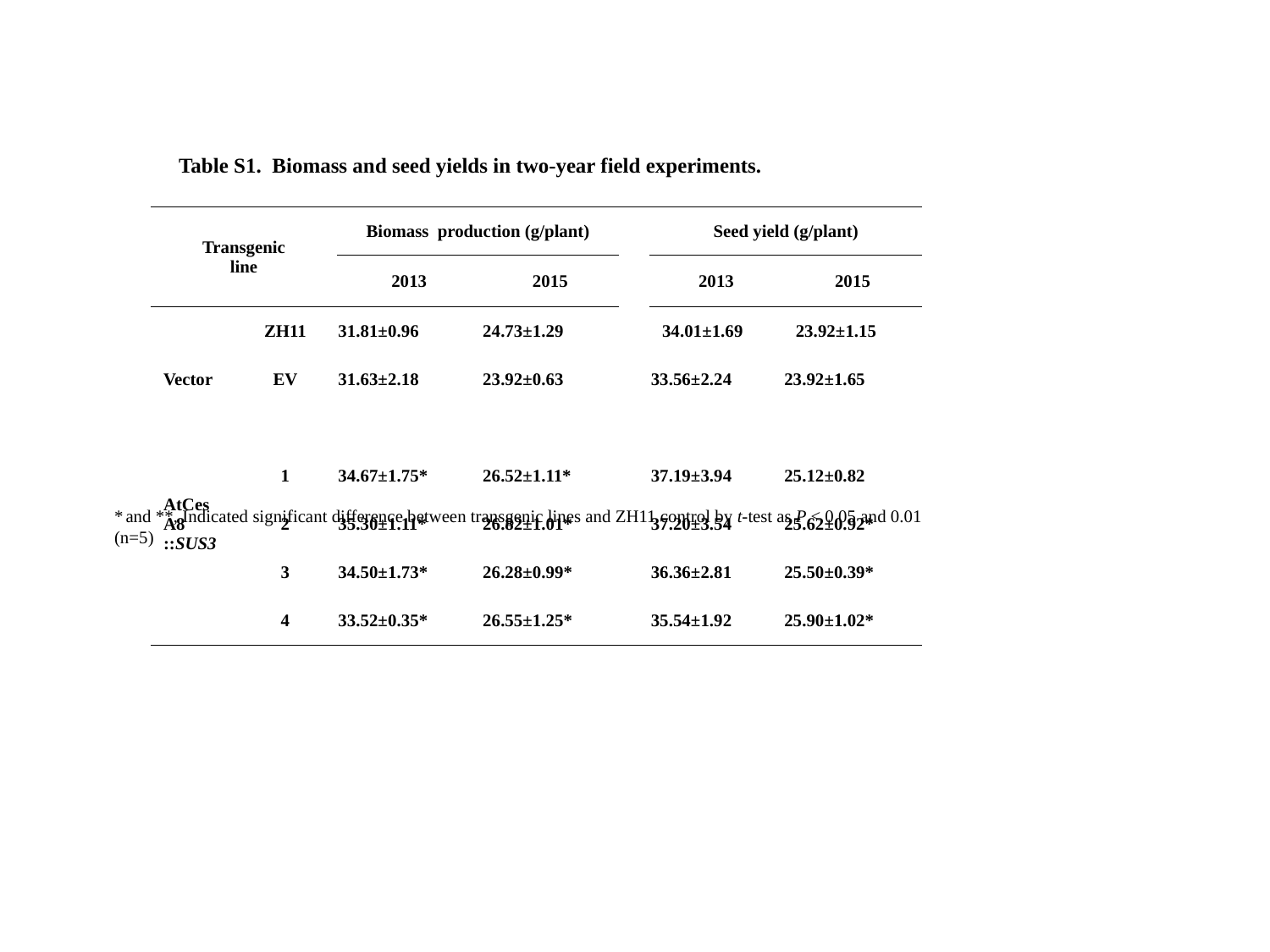

# Table S1. Biomass and seed yields in two-year field experiments.
| Transgenic line | | Biomass production (g/plant) | | | Seed yield (g/plant) | |
| --- | --- | --- | --- | --- | --- | --- |
| | | 2013 | 2015 | | 2013 | 2015 |
| | ZH11 | 31.81±0.96 | 24.73±1.29 | | 34.01±1.69 | 23.92±1.15 |
| Vector | EV | 31.63±2.18 | 23.92±0.63 | | 33.56±2.24 | 23.92±1.65 |
| | | | | | | |
| AtCesA8 ::SUS3 | 1 | 34.67±1.75\* | 26.52±1.11\* | | 37.19±3.94 | 25.12±0.82 |
| | 2 | 35.30±1.11\* | 26.82±1.01\* | | 37.20±3.54 | 25.62±0.92\* |
| | 3 | 34.50±1.73\* | 26.28±0.99\* | | 36.36±2.81 | 25.50±0.39\* |
| | 4 | 33.52±0.35\* | 26.55±1.25\* | | 35.54±1.92 | 25.90±1.02\* |
* and **, Indicated significant difference between transgenic lines and ZH11 control by t-test as P < 0.05 and 0.01 (n=5)

## Slide 4
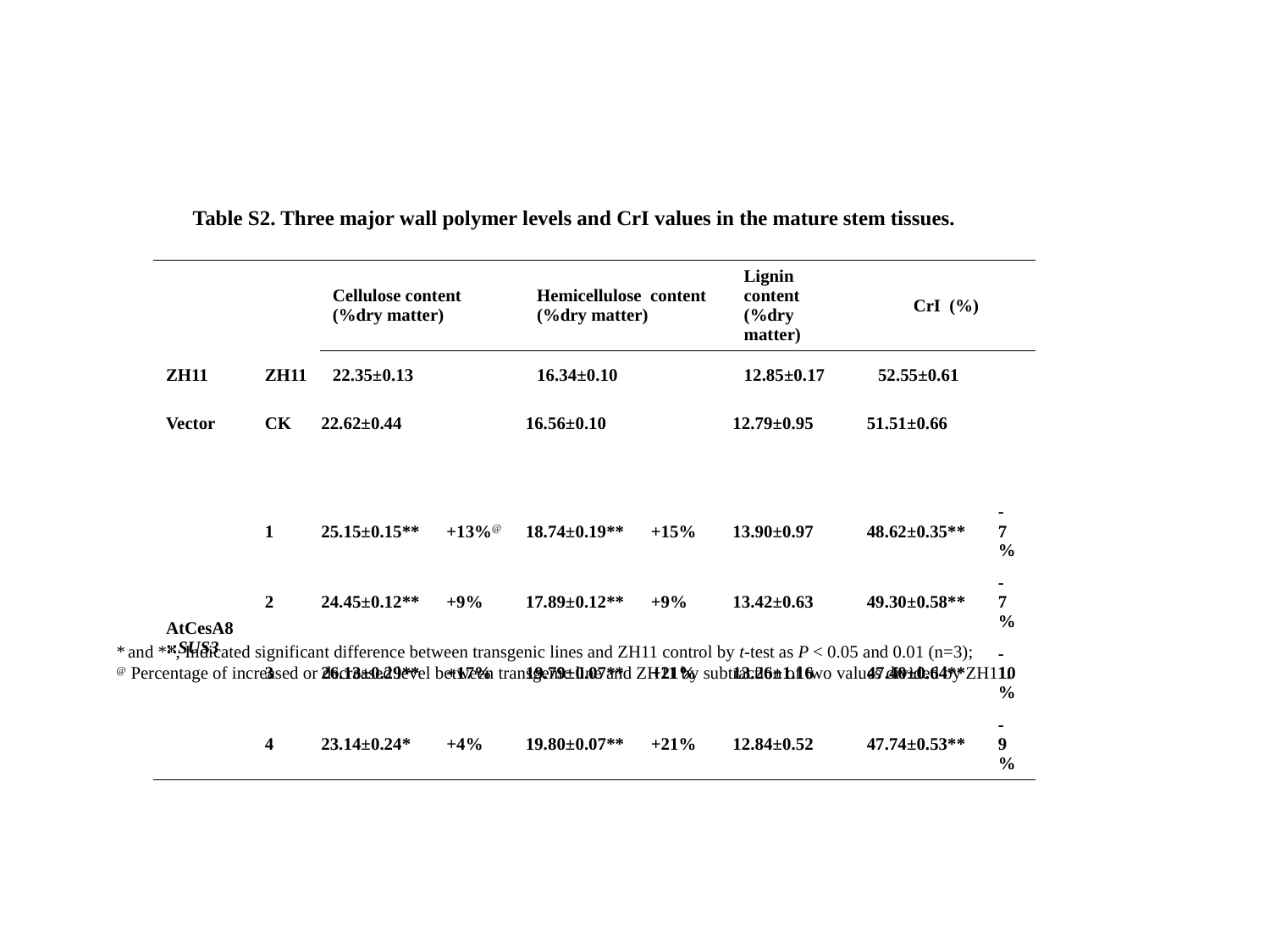

# Table S2. Three major wall polymer levels and CrI values in the mature stem tissues.
| | | Cellulose content (%dry matter) | | Hemicellulose content (%dry matter) | | Lignin content (%dry matter) | CrI (%) | |
| --- | --- | --- | --- | --- | --- | --- | --- | --- |
| ZH11 | ZH11 | 22.35±0.13 | | 16.34±0.10 | | 12.85±0.17 | 52.55±0.61 | |
| Vector | CK | 22.62±0.44 | | 16.56±0.10 | | 12.79±0.95 | 51.51±0.66 | |
| | | | | | | | | |
| AtCesA8 ::SUS3 | 1 | 25.15±0.15\*\* | +13%@ | 18.74±0.19\*\* | +15% | 13.90±0.97 | 48.62±0.35\*\* | -7% |
| | 2 | 24.45±0.12\*\* | +9% | 17.89±0.12\*\* | +9% | 13.42±0.63 | 49.30±0.58\*\* | -7% |
| | 3 | 26.13±0.29\*\* | +17% | 19.79±0.07\*\* | +21% | 13.26±1.16 | 47.40±0.64\*\* | -10% |
| | 4 | 23.14±0.24\* | +4% | 19.80±0.07\*\* | +21% | 12.84±0.52 | 47.74±0.53\*\* | -9% |
* and **, Indicated significant difference between transgenic lines and ZH11 control by t-test as P < 0.05 and 0.01 (n=3);
@ Percentage of increased or decreased level between transgenic line and ZH11 by subtraction of two values divided by ZH11.

## Slide 5
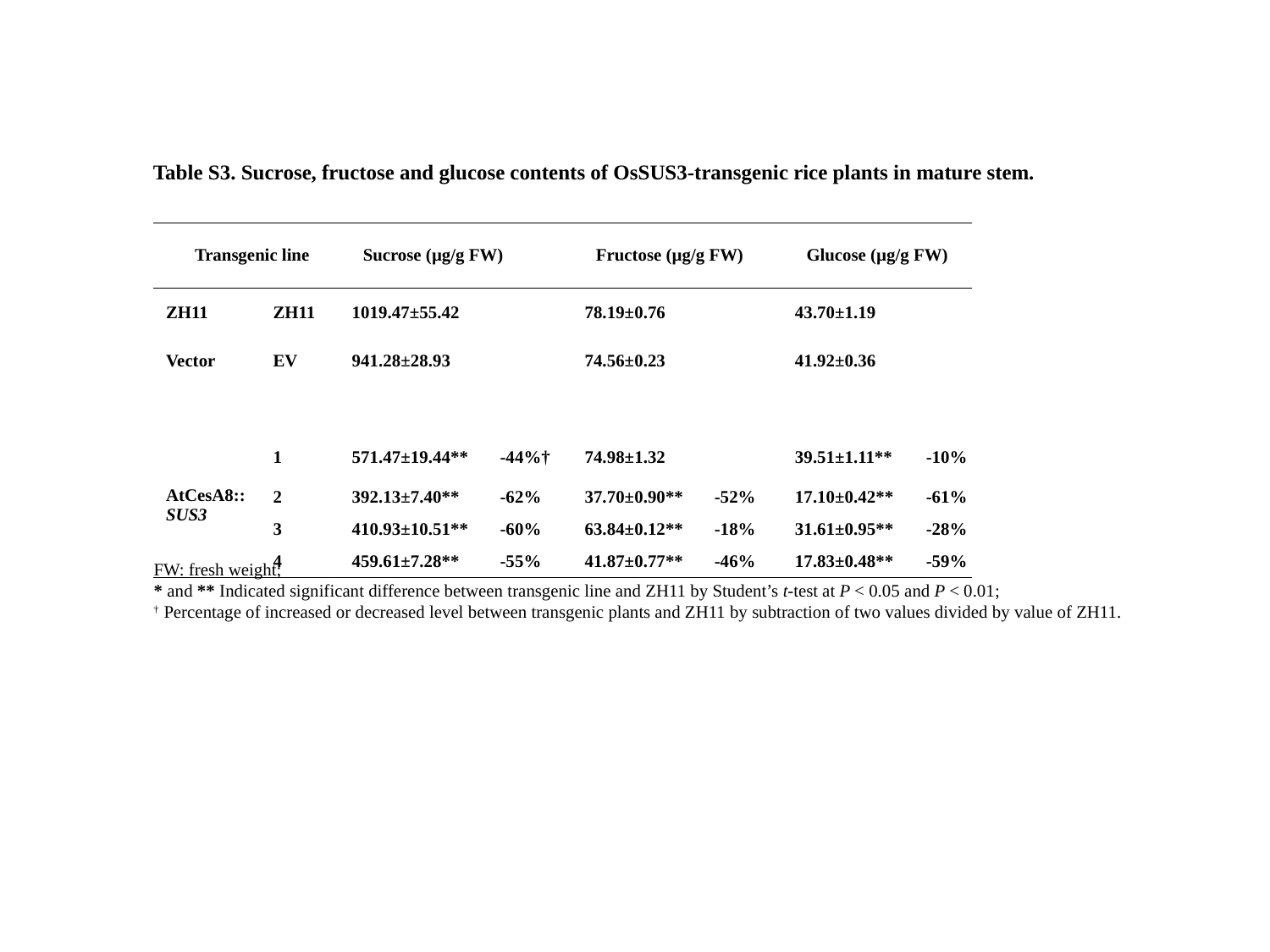

# Table S3. Sucrose, fructose and glucose contents of OsSUS3-transgenic rice plants in mature stem.
| Transgenic line | | Sucrose (µg/g FW) | | Fructose (µg/g FW) | | Glucose (µg/g FW) | |
| --- | --- | --- | --- | --- | --- | --- | --- |
| ZH11 | ZH11 | 1019.47±55.42 | | 78.19±0.76 | | 43.70±1.19 | |
| Vector | EV | 941.28±28.93 | | 74.56±0.23 | | 41.92±0.36 | |
| | | | | | | | |
| AtCesA8:: SUS3 | 1 | 571.47±19.44\*\* | -44%† | 74.98±1.32 | | 39.51±1.11\*\* | -10% |
| | 2 | 392.13±7.40\*\* | -62% | 37.70±0.90\*\* | -52% | 17.10±0.42\*\* | -61% |
| | 3 | 410.93±10.51\*\* | -60% | 63.84±0.12\*\* | -18% | 31.61±0.95\*\* | -28% |
| | 4 | 459.61±7.28\*\* | -55% | 41.87±0.77\*\* | -46% | 17.83±0.48\*\* | -59% |
FW: fresh weight;
* and ** Indicated significant difference between transgenic line and ZH11 by Student’s t-test at P < 0.05 and P < 0.01;
† Percentage of increased or decreased level between transgenic plants and ZH11 by subtraction of two values divided by value of ZH11.

## Slide 6
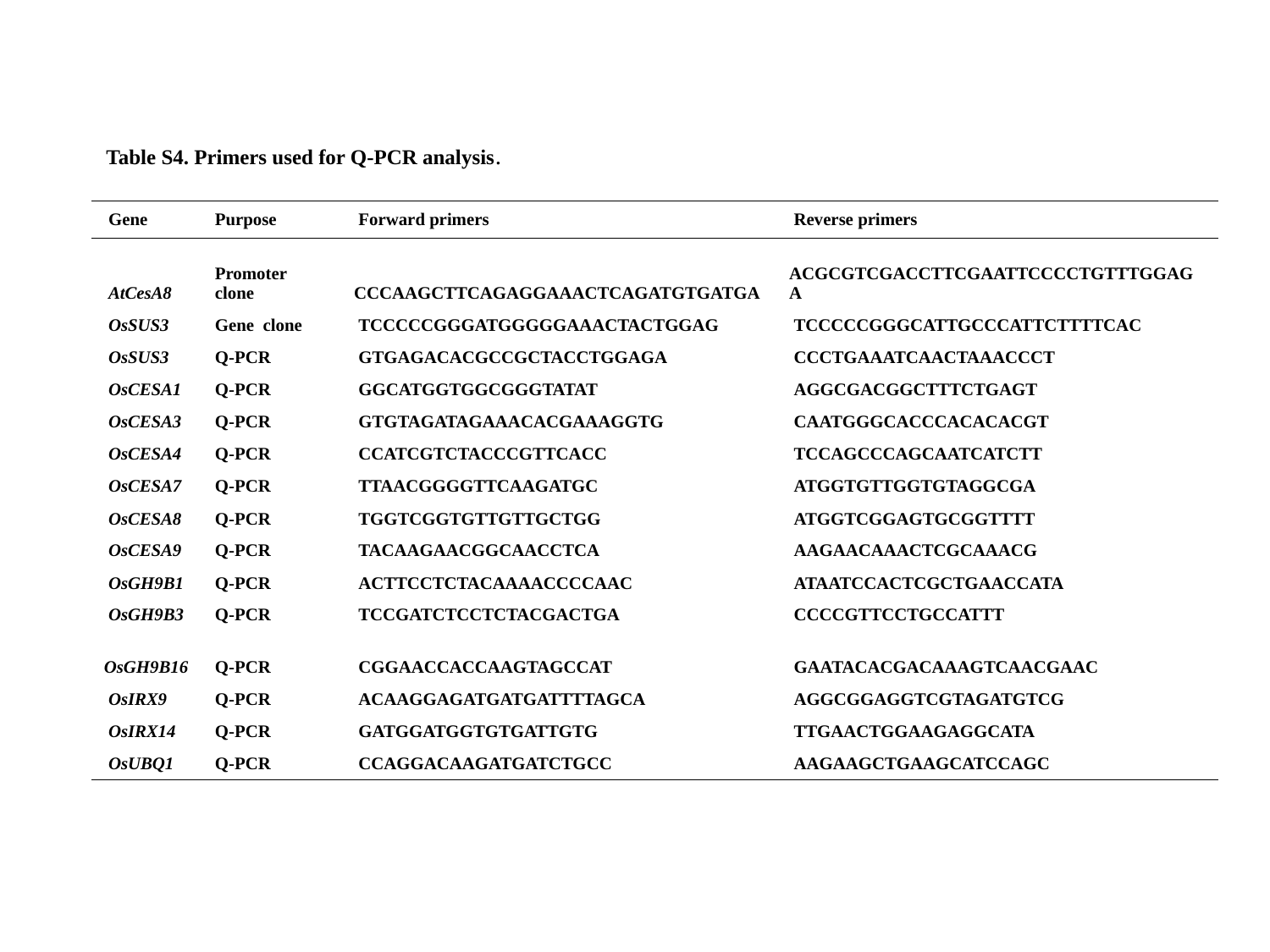

Table S4. Primers used for Q-PCR analysis.
| Gene | Purpose | Forward primers | Reverse primers |
| --- | --- | --- | --- |
| AtCesA8 | Promoter clone | CCCAAGCTTCAGAGGAAACTCAGATGTGATGA | ACGCGTCGACCTTCGAATTCCCCTGTTTGGAGA |
| OsSUS3 | Gene clone | TCCCCCGGGATGGGGGAAACTACTGGAG | TCCCCCGGGCATTGCCCATTCTTTTCAC |
| OsSUS3 | Q-PCR | GTGAGACACGCCGCTACCTGGAGA | CCCTGAAATCAACTAAACCCT |
| OsCESA1 | Q-PCR | GGCATGGTGGCGGGTATAT | AGGCGACGGCTTTCTGAGT |
| OsCESA3 | Q-PCR | GTGTAGATAGAAACACGAAAGGTG | CAATGGGCACCCACACACGT |
| OsCESA4 | Q-PCR | CCATCGTCTACCCGTTCACC | TCCAGCCCAGCAATCATCTT |
| OsCESA7 | Q-PCR | TTAACGGGGTTCAAGATGC | ATGGTGTTGGTGTAGGCGA |
| OsCESA8 | Q-PCR | TGGTCGGTGTTGTTGCTGG | ATGGTCGGAGTGCGGTTTT |
| OsCESA9 | Q-PCR | TACAAGAACGGCAACCTCA | AAGAACAAACTCGCAAACG |
| OsGH9B1 | Q-PCR | ACTTCCTCTACAAAACCCCAAC | ATAATCCACTCGCTGAACCATA |
| OsGH9B3 | Q-PCR | TCCGATCTCCTCTACGACTGA | CCCCGTTCCTGCCATTT |
| OsGH9B16 | Q-PCR | CGGAACCACCAAGTAGCCAT | GAATACACGACAAAGTCAACGAAC |
| OsIRX9 | Q-PCR | ACAAGGAGATGATGATTTTAGCA | AGGCGGAGGTCGTAGATGTCG |
| OsIRX14 | Q-PCR | GATGGATGGTGTGATTGTG | TTGAACTGGAAGAGGCATA |
| OsUBQ1 | Q-PCR | CCAGGACAAGATGATCTGCC | AAGAAGCTGAAGCATCCAGC |
